# Surface Modification Design for Improving the Strength and Water Vapor Permeability of Waterborne Polymer/SiO_2_ Composites: Molecular Simulation and Experimental Analyses

**DOI:** 10.3390/polym12010170

**Published:** 2020-01-09

**Authors:** Yingke Wu, Jianzhong Ma, Chao Liu, Hongxia Yan

**Affiliations:** 1 School of Materials Science & Engineering, Shaanxi University of Science and Technology, Xi’an 710021, China; einske@163.com; 2 Key Laboratory of Leather Cleaner Production, China National Light Industry, College of Bioresources Chemical and Materials Engineering, Shaanxi University of Science and Technology, Xi’an 710021, China; 3 Shaanxi Collaborative Innovation Center of Industrial Auxiliary Chemistry and Technology, Xi’an 710021, China; 4 Department of Applied Chemistry, School of Science, Northwestern Polytechnical University, Xi’an 710129, China; hongxiayan@nwpu.edu.cn

**Keywords:** interfacial interaction, molecular dynamics simulation, composites, surface modification

## Abstract

Polymer-based nanocomposites properties are greatly affected by interfacial interaction. Polyacrylate nanocomposites have been widely studied, but few studies have been conducted on their interface mechanism. Therefore, there was an urgent demand for providing a thorough understanding of the polymethyl acrylate/SiO_2_ (PMA/SiO_2_) nanocomposites to obtain the desired macro-performance. In this paper, a methodology, which combined molecular dynamics simulation with experimental researches, was established to expound the effect of the surface structure of SiO_2_ particles which were treated with KH550, KH560 or KH570 (KH550-SiO_2_, KH560-SiO_2_ and KH570-SiO_2_) on the mechanical characteristic and water vapor permeability of polymethyl acrylate/SiO_2_ nanocomposites. The polymethyl acrylate/SiO_2_ nanocomposites were analyzed in binding energy and mean square displacement. The results indicate that PMA/KH570-SiO_2_ had the highest tensile strength, while PMA/KH550-SiO_2_ had the highest elongation at break at the same filler content; KH550-SiO_2_ spheres can significantly improve water vapor permeability of polyacrylate film.

## 1. Introduction

Nanoparticles have received significant attention from researchers in the fields of electronics, metallurgy, aerospace, chemical engineering, biology and medicine due to their unique properties [1,2,3,4,5,6,7,8,9,10]. However, on account of the high surface energy of nanoparticles and the fact that they usually do not contain active functional groups, the dispersion of nanoparticles in polymers is very poor, thus leading to weak reinforcing effects [11,12]. A number of studies have shown that most properties of composite materials depend on the nanoparticles dispersion in polymer matrix and their interfacial interaction [13,14,15]. Therefore, there are numerous examples have been proposed for improving the dispersion of nanoparticles in polymer matrix thus improving the interfacial interactions by grafting or surface modified materials on the surface of nanoparticles for functionalization [16,17,18]. Meanwhile, effects of surface structure of nanomaterials on the properties of composites are still the focus of research [19,20,21].

Polyacrylate due to its excellent film forming properties, light stability, weather resistance and compatibility is widely used as a coating material for leather, textile materials, wood, metal surfaces, etc. However, the linear molecular structure and side chain polar groups always cause negative effects on mechanical properties, water resistance and water vapor permeability of the as-obtained films, which limits its scope of use. With the development of nanotechnology, many nanoparticles have been attempted to introduce into polyacrylate to improve its film-forming properties. Chen et al. synthesized polyacrylate/modified-TiO_2_ coating film [22]. The results prove that the Modification of TiO_2_ particles can build up their dispersibility in polyacrylate coating and fall off water absorption of coating. Zhao et al. prepared an organic nano-SiO_2_/fluorinated polyacrylate composite latex [23]. The results showed that the films exhibit strong hydrophobicity. From our previous studies, Pickering emulsion steadied by silica sol has fine effects on pigment printing adhesives [24]. The improvement of latex film performance is due to the addition of nano-TiO_2_ [25]. We have done a lot of research on polyacrylate/silica [26,27,28,29]. The results show that the introduction of silica improves the mechanical properties, thermal properties and water vapor permeability of polyacrylate film. However, intensive studies on the interfacial interaction between polyacrylate and SiO_2_ particles have rarely been reported. The interaction between polymers and SiO_2_ particles originate from molecules, atoms, and their underlying quantum mechanical arguments (nanoscopic and/or sub-nanometer), which interactions are hard to observe and examine by traditional experimental tests.

A strong supporting tool for studying the interfacial interactions between the polymer matrix and nanoparticles is molecular dynamics (MD) simulations, which has been diffusely used to view the interaction machine-processed between different materials [30,31,32,33,34,35,36,37]. Rissanou et al. analyzed several graphene/polymer nanocomposites by atomistic molecular dynamics simulations [38]. The results indicate that chain segmental dynamics is slower at the PE/graphene interface than the bulk one. Wang et al. have performed the mechanical properties of PET/silica composites by MD simulations in detail [39]. The simulation results shown that nanocomposites have the higher mechanical properties in comparison with those in pure PET system, ascribing a stronger interaction between the modified silica and polymer chains. We have successfully employed MD simulation to check on the presence of p-p stacking interaction between poly (styrene-butyl acrylate) latex (P(St-BA)) and sulfonated graphene nanosheet (S-GNS) [40].

In this work, we study the effects of interfacial structure between SiO_2_ particle and polymer matrix on the properties of their composites. SiO_2_ was modified by polysiloxane (KH550, KH560 and KH570) with similar chain length to enhance its interfacial compatibility and binding with PA. The properties of its composites were studied through experiments and MD to explore its influence rule. This study is expected to provide a theoretical basis for the structural design of nanoparticles and their applications in functional composites.

## 2. Materials and Methods

### 2.1. Materials

All chemicals were of analytical grade and used without any further purification. Octyltrimethoxysilane (OTMS, 97%), ammonia, ethanol, tetraethoxysilane (TEOS), 3-aminopropyl triethoxy silane (KH550, 97%), γ-glycidoxypropyl trimethoxy silane (KH560, 97%), γ-methacryloxypropyl trimethoxy silane (KH570, 97%), xylene, methyl acrylate (MA), sodium dodecyl sulfate (SDS) and potassium persulfate (KPS) were all purchased from Tianjin Fuchen Chemical Reagent Factory.

### 2.2. Preparation of SiO_2_ Nanoparticles and Modified-SiO_2_ Nanoparticles

The procedure for preparing SiO_2_ nanoparticles was as follows: firstly, 5 mL of ammonia, 100 mL of ethanol and 5mL of TEOS were added into 250 mL three-necked flask. Then, the mixture was stirred for 5 h at 60 °C. Finally, the product was centrifuged and washed by deionized water and ethanol for several times, then dried for 12 h at 60 °C to produce a white SiO_2_ powder.

The procedure for preparing SiO_2_ nanoparticles modified by KH570 as follows:

About 5 mL ethanol solution of KH570 was gradually added to 100 mL SiO_2_ nanoparticles ethanol dispersion under a magnetic stirrer. Then, the mixed solution was stirred at room temperature for 72 h. The mixed solution was centrifuged and washed with xylene, ethanol and deionized water several times, then SiO_2_ nanoparticles modified by KH570 were attained via drying for 12 h at 60 °C (KH570-SiO_2_).

The procedure for preparing SiO_2_ nanoparticles modified by KH550, which is defined as KH550-SiO_2_ (SiO_2_ modified by KH560, which is defined as KH560-SiO_2_) as follows: SiO_2_ nanoparticles were ultrasonic dispersed into 100 mL of ethyl alcohol at 25 °C for 30 min, and the pH of the mixed solution dispersion reached 6 with glacial acetic acid. Then 5 mL of KH550 (or KH560) was added into the solution under the stirring at 60 °C for 6 h. The obtained solution was then centrifuged and washed by xylene, ethanol and deionized water for several times, and dried at 60 °C for 6 h to obtain KH550-SiO_2_ (or KH560-SiO_2_) nanoparticles.

### 2.3. Preparation of PMA/Modified-SiO_2_ Nanocomposite and its Composite Film

Some modified-SiO_2_ (2 wt% of PMA mass) and SDS were ultrasonic dispersed for 10 min at 75 °C. Then KPS and MA were added to the emulsion drop wisely, while the polymerization was conducted at 75 °C for 6 h.

Finally, the PMA/modified-SiO_2_ composite emulsion was poured into polytetrafluoroethylene (PTFE) mold and laid on the horizontal surface until dried completely at room temperature to obtain PMA/modified-SiO_2_ composite film. PMA/SiO_2_ composite film was prepared in the same method.

### 2.4. Characterization and Measurements

The microstructures and morphology of all samples were measured by scanning electron microscope (SEM, S4800, Rigaku) and transmission electron microscope (TEM, Tecnai G2 F20, FEI). The chemical structures of all materials were analyzed by fourier transform infrared spectrum (FT-IR, VECTOR-22, Brucker) and Ultraviolet-visible-near infrared spectrophotometer (Cary 5000, Agilent). The thermal stability of the samples was studied by thermogravimetric analysis (TGA, STA409PC, Netzsch) at the nitrogen atmosphere from room temperature to 600 °C with a heating rate of 5 °C/min. The glass transition process of samples was characterized using a differential scanning calorimeter (DSC, Q5000 IR) The mechanical properties were tested by a servo material multi-functional high and low temperature control testing machine (AI-7000-NGD, Goodtechwill) at a loading rate of 100 mm/min according to QB/T 1331-1998. The water vapor transmission rate (WVP) was tested by a Water vapor transmittance tester (W3/060, Labthink) according to QB/T 1279-2012.

### 2.5. Simulation Methodologies

The reactant molecules were built in the Visualizer module of Material Studio 8.0 software (Accelrys Inc., San Diego, CA, USA). For the MD simulations, the Forcite and Amorphous cell modules of the Materials Studio suite of software were used. All the theoretical calculations were performed using the Condensed-Phase Optimized Molecular Potentials for Atomistic Simulation Studies COMPASS force field [41,42].

#### 2.5.1. Construction of SiO_2_ Nanoparticles

The xsd molecular model of SiO_2_ is imported from MS software material library.

**Figure 1:**
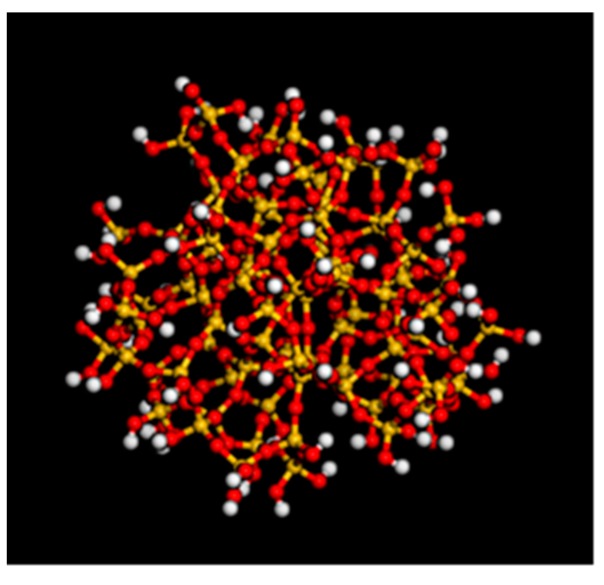
SiO_2_ nanoparticle without broken bonds on the surface.

After the unit cell model of silica is obtained, it is geometrically optimized to obtain a lower energy structure. Next, the silica particles with a radius of 1 nm (10 Å) are constructed, and a spherical silica nanoparticle with a surface saturated with unsaturated bonds between Si atoms and O atoms is obtained. Select the broken bond on the Si atom on the surface of the SiO_2_ unit cell and combine it with -OH, and combine the broken bond on the surface O atom with the H atom. Increase the reliability, and optimize the structure to obtain a spherical SiO_2_ model (Figure 1).

**Figure 2:**
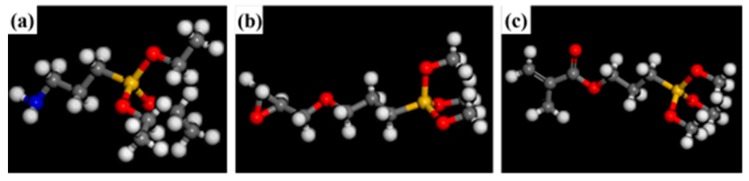
Molecular model of modifiers: (**a**). KH550, (**b**). KH560, (**c**). KH570 (Gray represents C atom, yellow represents Si atom, white represents H atom, red represents O atom and blue represents N atom).

**Figure 3:**
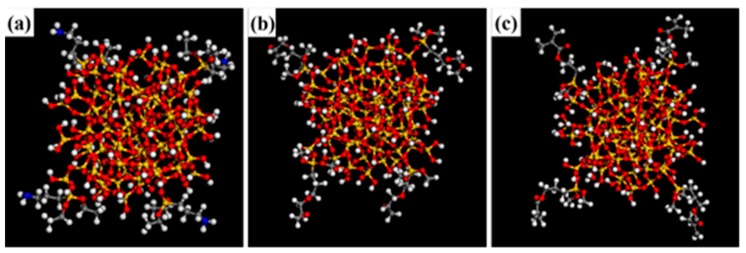
Model of modified-SiO_2_: (**a**). KH550-SiO_2_, (**b**). KH560- SiO_2_, (**c**). KH570-SiO_2_.

Three different modifiers were grafted on the silica surface, and the three modifiers were KH550 (2a), KH560 (2b) and KH570 (2c). The structure of the modified surface was optimized, and the energy converged to 1 × 10^−4^ kca1/mol. Figure 2 shows the structure of three modifiers, and Figure 3 shows the surface of modified-SiO_2_. In this paper, four identical silane coupling agent chains were grafted onto SiO_2_ sphere, and the grafted microspheres were optimized by the Smart method to optimize the energy to 1 × 10^−4^ kca1/mol. In order to search for the optimal structure, the cell is then annealed at 0.1 MPa from the low temperature of 300 K to the upper temperature of 500 K for 200 ps to prevent the system to form being trapped at a local high energy minimum. Subsequently, 200 ps of NVT (constant number of particles, volume, and temperature) simulation is performed at 298 K.

**Figure 4:**
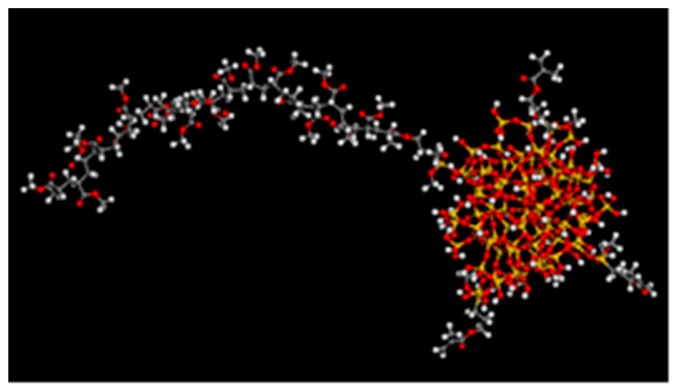
Model of PMA-KH570-SiO_2_.

Since the double bond on KH570-SiO_2_ is polymerized with MA, in this paper a model of polymerizing one double bond on the surface of KH570-SiO_2_ with MA (PMA-KH570-SiO_2_) is constructed. PMA polymer chain has 20 repeat units, as shown in the Figure 4.

#### 2.5.2. Construct the Composite System Model

PMA/SiO_2_ (KH550-SiO_2_, KH560-SiO_2_): Amorphous cells containing composites of PMA polymer chains with 20 repeat units and a SiO_2_ (or modified-SiO_2_) nanoparticle (diameter 20 nm) were constructed, and periodic boundary conditions were applied.

PMA/KH570-SiO_2_: Amorphous cells containing composites of PMA polymer chains with 19 repeat units and one PMA-KH570-SiO_2_ (Figure 4) were constructed, and periodic boundary conditions were applied.

To study the diffusion coefficient of H_2_O in composite systems, the MSDs of H_2_O in composite systems were analyzed. Some composite systems containing water molecules were constructed as follows (Supplementary Materials, Figure S1):

PMA/SiO_2_ (KH550-SiO_2_, KH560-SiO_2_)/H_2_O: Amorphous cells containing composites of PMA polymer chains with 20 repeat units, one SiO_2_ (or modified-SiO_2_) nanoparticle (diameter 20 nm) and 10 H_2_O molecules were constructed, and periodic boundary conditions were applied.

PMA/KH570-SiO_2_/H_2_O: Amorphous cells containing composites of PMA polymer chains with 19 repeat units, one PMA-KH570-SiO_2_ (Figure 4) and 10 H_2_O molecules were constructed, and periodic boundary conditions were applied.

#### 2.5.3. Molecular Dynamics Simulation Process

**Figure 5:**
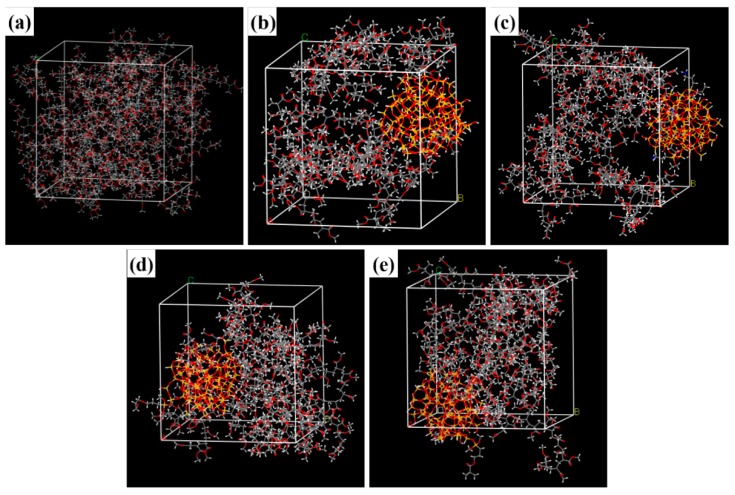
Models for MD simulation of composite system: (**a**). PMA, (**b**). PMA/SiO_2_, (**c**). PMA/KH550-SiO_2_, (**d**). PMA/KH560-SiO_2_, and (**e**). PMA/KH570-SiO_2_.

After building PMA/SiO_2_ (or modified-SiO_2_) composite systems, the energy of each generated cell is minimized to a convergence value of 1.0 × 10^−4^ kcal mol^−1^ by using the Smart Minimizer method to relax the state of minimal potential energy. Whereafter, 200 ps of NVT (constant number of particles, volume, and temperature) simulation is performed at 298 K. The cell is then annealed at 0.1 MPa from the low temperature of 300 K to the upper temperature of 500 K for 200 ps to prevent the system to form being trapped at a local high energy minimum [43]. Subsequently, 200 ps of NVT (constant number of particles, volume, and temperature) simulation is performed at 298 K, 500 ps of NPT (constant number of particles, pressure, and temperature) simulation is performed at 0.1 MPa and 2 ns of NVE (constant number of particles, volume, and energy) simulation is performed to further relax the polymer structure by using the Andersen Thermostat for temperature control and the Berendsen Barostat for pressure control [44,45] (Figure 5).

At last, the cell can be used to analyze properties of the system. In order to further verify the effect of the number of polymer chains on the properties of composites, a composite system with 30 polymer chains was studied (Figure S2). The results show that the binding energy between the polymer and SiO_2_ is not significantly different from the data of the composite system of 20 polymer chains in the article, which is reasonable (Tables S1 and S2). This result indicates that the composite system of 20 polymer chains may basically match the experiment. With the increasing of the number of polymer chains, the performance of the composite system has not changed significantly.

**Figure 6:**
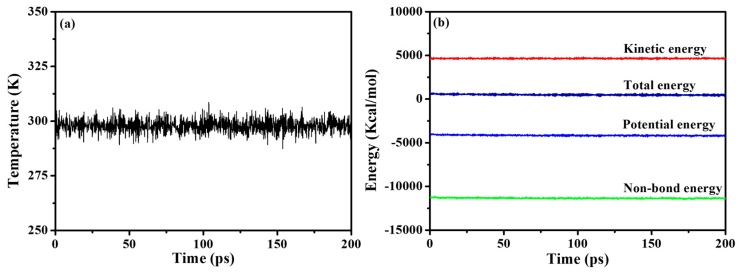
Temperature (**a**) and energy (**b**) of PMA/SiO_2_ nanocomposite at a temperature of 298K during the MD simulation.

System equilibrium is judged by temperature and energy balance. Figure 6 shows the trajectory temperature and energy fluctuation chart of 200 ps NVT in the MD equilibrium stage. From the Figure 5, the trajectory energy fluctuation of each frame is gentle, indicating that the system energy has reached equilibrium.

The above two criteria showed that the PMA/SiO_2_ composite system has indeed reached equilibrium through MD simulation, and the subsequent analysis results are reliable. The remaining PMA and modified-SiO_2_ interaction systems could all reach the same conclusion.

## 3. Results

### 3.1. Morphological and Structural Characterization of Modified-SiO_2_

**Figure 7:**
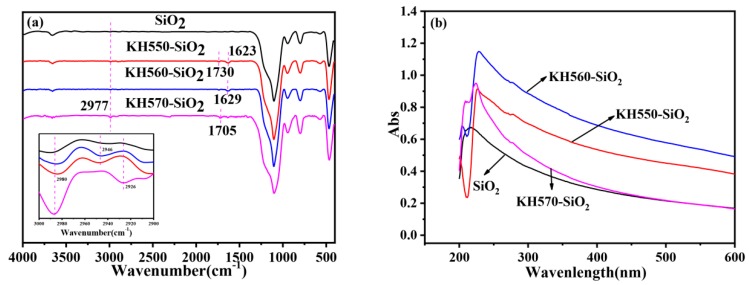
(**a**) FT-IR spectrum of SiO_2_ and modified-SiO_2_, (**b**) UV curve of SiO_2_ and modified-SiO_2_.

Surface modification is essential for the synthesis and functionality of composites. FT-IR is often used to characterize surface modification. FT-IR spectra of SiO_2_ and modified-SiO_2_ are shown in Figure 7a. The characteristic peaks assigned to the stretching vibration of Si-O-Si at 1101 cm^−1^ are observed in the spectrum of SiO_2_. After modification with silane coupling agent, there appeared absorption peaks at 1705 cm^−1^ (C=C stretching vibration) in the spectrum of KH570-SiO_2_, as well as peaks at 1730 cm^−1^ (–C–H– asymmetric stretching vibration), 1623 cm^−1^ (N–H in-plane bending vibration) in the spectrum of KH550-SiO_2_. The peak at 2977 cm^−1^ reveals the existence of –CH_3_ or –CH_2_– on SiO_2_ surface. These may suggest that silane coupling agent is successfully grafted onto the SiO_2_ surface.

To reveal the chemically bonding between SiO_2_ and the silane coupling agent, UV absorption spectrum of the modified-SiO_2_ was characterized. It can be seen in Figure 7b that there is a small shift in the position of the absorption peak, which may be attributed to changes in the surface structure of SiO_2_.

**Figure 8:**
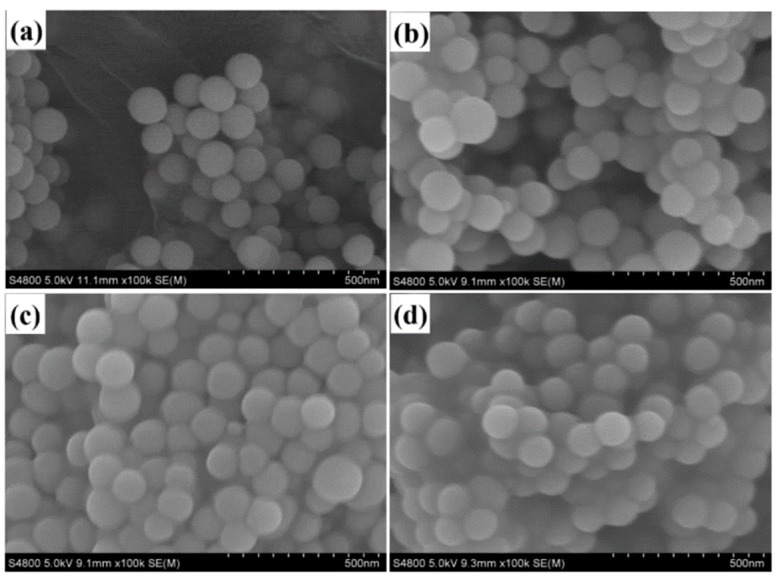
SEM images of nano-SiO_2_ (**a**). SiO_2_, (**b**). KH550-SiO_2_, (**c**). KH560-SiO_2_, (**d**). KH570-SiO_2_.

SEM images (Figure 8) present that the as-prepared SiO_2_ and modified-SiO_2_ samples are uniform in size with spherical shape, and the average size was about 80 nm. Meanwhile, SiO_2_ are well dispersed (See Figure 8a). Nevertheless, the modified-SiO_2_ are not particularly well dispersed (Figure 8b–d), which may be caused by self-polymerization of the silane coupling agent.

### 3.2. Morphological and Structural Characterization of PMA/SiO_2_ Composite Emulsion and Film

**Figure 9:**
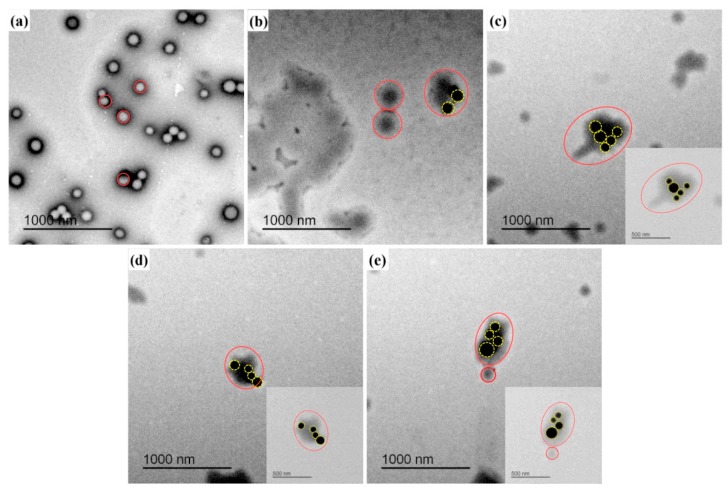
TEM images: (**a**) PMA latex, (**b**) PMA/SiO_2_ composite, (**c**) PMA/KH550-SiO_2_ composite, (**d**) PMA/KH560-SiO_2_ composite, and (**e**) PMA/KH570-SiO_2_ composite (Red circles refer to latex particles, yellow circles refer to SiO_2_ particles).

Figure 9 shows the TEM image of PMA and PMA/SiO_2_ composite latex particles. As shown in Figure 9a, the pristine PMA latex particles display a well-defined spherical morphology and the latex particle size is 100–200 nm. The black phase which are SiO_2_ particles are on the surface of latex particles (the fuzzy layers). SiO_2_ is located onto the surface of latex particles (Figure 9b), KH550-SiO_2_ is onto the surface of latex particles (Figure 9c), and KH560-SiO_2_ is also on the surface of latex particles (Figure 9d). There are more KH550-SiO_2_ and 560-SiO_2_ particles are on the surface of latex particles, this is mainly because that stronger hydrogen bonds or electrostatic interactions is formed between modified-SiO_2_ nanoparticles and latex particles, compared with SiO_2_ nanoparticles. KH570-SiO_2_ nanoparticles which enter the interior of PMA latex particles seem less clear (Figure 9e), and other nanoparticles appear on the latex particle surface as black phase [46]. Different surface modification between the above four SiO_2_ sources explains the difference in the distribution of SiO_2_ nanoparticles.

**Figure 10:**
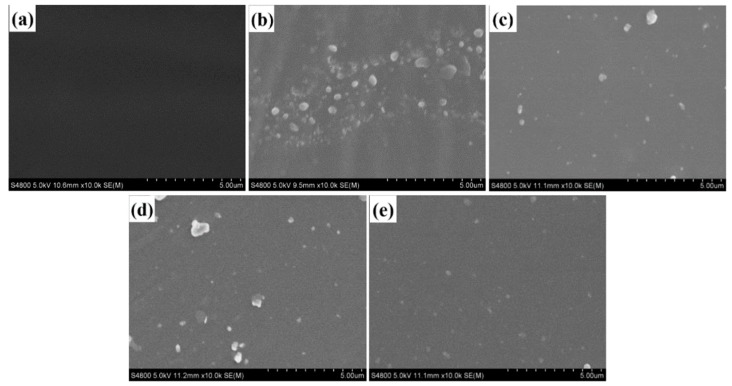
SEM of composite films: (**a**) PMA, (**b**) PMA/SiO_2_, (**c**) PMA/KH550-SiO_2_, (**d**) PMA/KH560-SiO_2_ and (**e**) PMA/KH570-SiO_2_).

The dispersion of SiO_2_ particles in the PMA film before and after modification can be observed by SEM, as shown in Figure 10. The untreated SiO_2_ particles aggregated severely in PMA film with the size equivalenting about to 500 nm (Figure 10b). The KH560-SiO_2_ particles have better dispersibility in PMA, and good interfacial adhesion with PMA film. Which is better than those of the unmodified particles (Figure 10c). Nevertheless, there are still some aggregates in PMA film. KH550-SiO_2_ particles are well dispersed as small aggregates (Figure 10d). When PMA film was filled with KH570-SiO_2_ particles, the nanoparticles are uniformly dispersed in PMA film, and it is difficult to see the aggregate of the nanoparticles (Figure 10e). The interfacial compatibility between KH570-SiO_2_ nanoparticles and PMA film is well.

The unmodified-SiO_2_ particles aggregate in PMA film on account of their high polar surface energy. The poor compatibility of aggregated SiO_2_ particles with the PMA film is due to the hydrophilic surface. When SiO_2_ particles are modified with KH560, their surface is covered by long alkyl chain, which gives the particles a well interfacial compatibility to PMA film [47]. The interface compatibility between KH550-SiO_2_ particles and PMA film is a bit better, which introduce the amidogen group. The KH570-SiO_2_ particles can form chemically bond with PMA matrix through double bond polymerization. Good compatibility makes better dispersion of SiO_2_ particles in PMA film.

### 3.3. Properties of PMA/SiO_2_ and PMA/Modified-SiO_2_ Composite Films

#### 3.3.1. Mechanical Properties of PMA/SiO_2_ and PMA/Modified-SiO_2_ Composite Films

**Figure 11:**
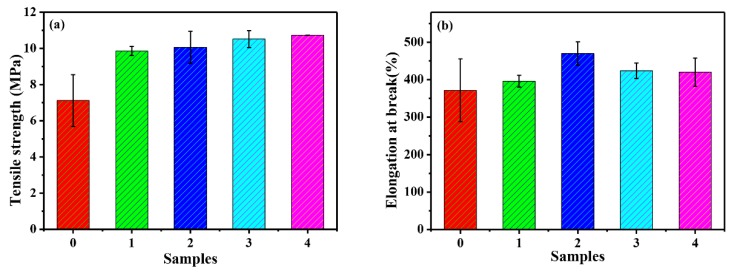
Effect of fillers on mechanical properties of composite films: (**a**) Tensile strength, (**b**) Elongation at break (**0.** PMA, **1.** PMA/SiO_2_, **2.** PMA/KH550-SiO_2_, **3.** PMA/KH560-SiO_2_ and **4.** PMA/KH570-SiO_2_).

Figure 11 reveals the tensile strength and elongation at break of PMA/SiO_2_ nanocomposites, in which SiO_2_ is modified by different silane coupling agents. As everyone knows, the interfacial interaction between polymer and nanoparticles has a greater influence on the tensile strength of composites [20]. The weak interfacial interaction between polymer and nanoparticles results in less stress being transferred from polymer to nanoparticles [47]. The stronger the interfacial interaction between polymer and nanoparticles, the greater the stress transmitted by the polymer to the nanoparticles, resulting in higher tensile strength. As can be seen in Figure 11a, the tensile strength of PMA/modified-SiO_2_ film is higher than that of PMA/SiO_2_ film. And, the tensile strength of PMA/KH570-SiO_2_ film have the highest value than that of other films.

The dispersion and interfacial interaction between polymer and nanoparticles have a great influence on elongation at break of nanocomposites [48,49]. Good dispersibility and proper interfacial interaction can enhance the value of elongation at break, while excessive strong interfacial interaction can reduce it. The elongation at break of composite films is shown in Figure 11b, compared with KH550-SiO_2_, the addition of KH570-SiO_2_ reduced the elongation at break of the film, which is consistent with the strong interfacial interaction between PMA and KH570-SiO_2_.

#### 3.3.2. Water Vapor Permeability of PMA/SiO_2_ and PMA/Modified-SiO_2_ Composite Films

Water vapor permeability is an important index when emulsion products are used in coatings requiring air permeability. The water vapor permeation rate is primarily assumed by the diffusion process and adsorption/desorption process, which are influenced by the composition and structure of the polymer chains. The soft polymer chains of polyacrylate can give more free volume for the passage of water vapor molecules, although hydrophobic segments of polyacrylate are detrimental to the adsorption process [50].

**Figure 12:**
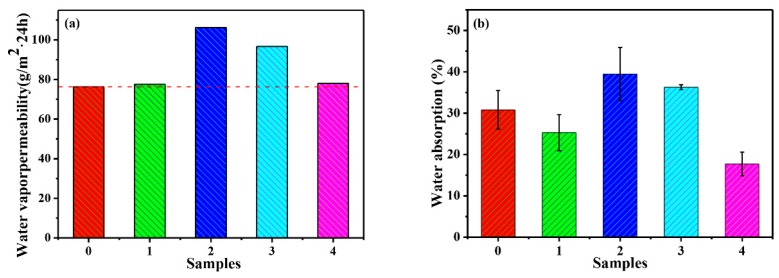
(**a**) Effects of fillers on water vapor permeability and (**b**) water resistance of composite films (**0.** PMA, **1.** PMA/SiO_2_, **2.** PMA/KH550-SiO_2_, **3.** PMA/KH560-SiO_2_ and **4.** PMA/KH570-SiO_2_).

The effects of functionalized SiO_2_ on water vapor permeability of as-obtained films are shown in Figure 12a. Compared with pure PMA film (Figure 12a), water vapor transmission rate of composite films is improved. It is evident that by blending the KH550-SiO_2_ or KH560-SiO_2_ nanoparticles in the PMA film, water vapor permeability of PMA/SiO_2_ nanocomposite films increases significantly. This is mainly attributed to the fact that KH550-SiO_2_ and KH560-SiO_2_ contain a hydrophilic amino group or an epoxy group to facilitate water vapor transmission through the film. It can be seen that the water vapor transmission rate of PMA/SiO_2_ and PMA/KH570-SiO_2_ composite films are higher than that of PMA composite film, which is mainly result from that: On the one hand, there is a large number of interfacial pores between SiO_2_ nanoparticles and PMA film, which provides a good channel for water vapor molecules. On the other hand, an enhancement in the amount of hydrophilic groups in film leads to an increase in water vapor permeability [51]. The surface of SiO_2_ nanoparticles contains a large amount of hydroxyl groups, which increases the number of hydrophilic groups inside the film.

#### 3.3.3. Water Resistance of PMA/SiO_2_ and PMA/Modified-SiO_2_ Composite Films

Under normal circumstances, water absorption of the composite film is used to reflect its water resistance, and the higher the water absorption rate, the worse the water resistance. It can be seen from Figure 12b that compared with PMA film, water absorption of PMA/KH550-SiO_2_ and PMA/KH560-SiO_2_ composite films show higher value than that of PMA. While water absorption of PMA/SiO_2_ and PMA/KH570-SiO_2_ composite films show lower value than those of PMA, and the PMA/KH570-SiO_2_ composite film has the lowest water absorption. This is mainly due to the fact that KH550-SiO_2_ and KH560-SiO_2_ contain a hydrophilic amino group and an epoxy group to facilitate water vapor transmission through the film, while PMA/KH570-SiO_2_ contains a hydrophobic C=C which is not favorable for water vapor transmission through the film.

#### 3.3.4. Thermal Properties of PMA/SiO_2_ Composite Films

**Figure 13:**
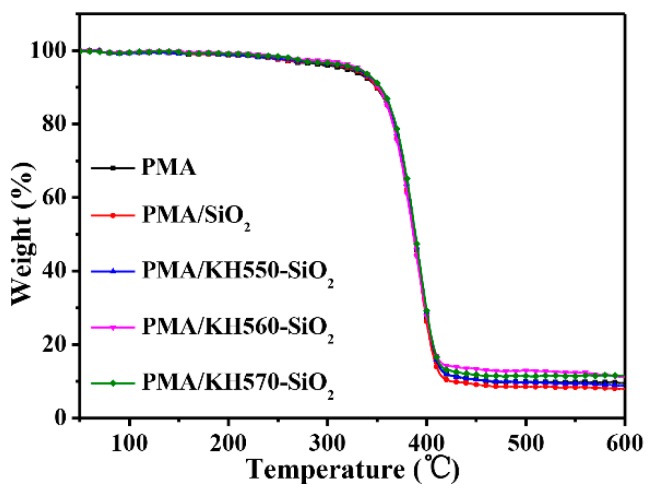
TGA curve of composite films.

TGA curves of as-prepared films are shown in Figure 13. Compared with PMA film, the T_5_ of PMA/SiO_2_ and PMA/modified-SiO_2_ increased.

The corresponding characteristic heat data for all samples is shown in Table 1. By comparing, heat-resistance index of the PMA/SiO_2_ and PMA/modified-SiO_2_ composite films obviously increased. This is mainly because that the addition of SiO_2_ nanoparticles causes entanglement of polymer chain, which slows down the decomposition of the molecular chain. The heat-resistance index of PMA/modified-SiO_2_ composite films is higher than that of PMA/SiO_2_. In addition, the heat-resistance index of PMA/KH570-SiO_2_ composite film is the highest. This is mainly ascribed to the stronger interface interaction between PMA and KH570-SiO_2_ [52,53].

### 3.4. Molecular Dynamics Simulation

#### 3.4.1. Binding Energy Analysis

Molecular dynamics simulations are currently effective methods for verifying the strength of interfacial interactions. The strength of the interaction between the PMA film and the SiO_2_ particles can be reflected by the amount of binding energy between them. Generally, the greater the binding energy, the stronger the interaction force between PMA film and SiO_2_ particles. As a result, the simulation of the binding energy between PMA film and SiO_2_ (or modified-SiO_2_) particles can be used to study the interaction mechanism. The binding energies of PMA/SiO_2_ and PMA/modified-SiO_2_ composite materials can be used by:

(1) $$E_{bingding}=-E_{\mathrm{inter}}=-(E_{total}-E_{PMA}-E_{SiO_{2}}),E_{bingding}=-E_{\mathrm{inter}}=-(E_{total}-E_{PMA}-E_{modified-}{}_{SiO_{2}})$$

where *E_total_* is the energy of the PMA/SiO_2_ or PMA/modified-SiO_2_, *E_PMA_* is the energy of PMA, and *E_Si_*_*O*_2__ is the energy of SiO_2_ particles, *E_modified-Si_*_*O*_2__ is the energy of modified-SiO_2_ particles. The binding energies between PMA and SiO_2_ (or modified-SiO_2_) are given in Table 2.

The total energy of the PMA/SiO_2_ (modified-SiO_2_) system, the energy of PMA, and the energy of SiO_2_ (modified-SiO_2_) are presented in Table 2.

The binding energy of PMA/KH550-SiO_2_ reaches a higher value than PMA/SiO_2_, showing the strongest interfacial interaction between PMA film and KH550-SiO_2_ particles. A higher binding energy shows good compatibility between PMA film and KH550-SiO_2_ particles. As the KH560-SiO_2_ was added into PMA, the binding energy of the PMA/KH560-SiO_2_ film is better than that of PMA/KH550-SiO_2_ film, a sign of well compatibility of PMA film with KH560-SiO_2_ particles [54].

#### 3.4.2. MSD (Mean Square Displacement) and Diffusion Coefficient (D) of Water in Composite System

**Figure 14:**
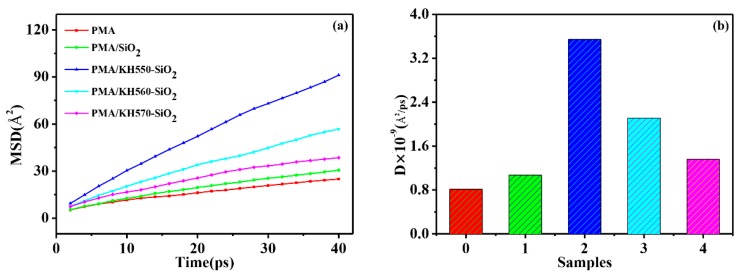
(**a**) MSD diagrams of H_2_O in the PMA, PMA/SiO_2_, PMA/KH550-SiO_2_, PMA/KH560-SiO_2_, and PMA/KH570-SiO_2_ films during the 40-ps MD duration, (**b**) The diffusion coefficient (D) of H_2_O in composite system: **0.** PMA, **1.** PMA/SiO_2_, **2.** PMA/KH550-SiO_2_, **3.** PMA/KH560-SiO_2_, and **4.** PMA/KH570-SiO_2_.

The diffusion coefficient (D) of water molecules in nanocomposite reflects the water vapor permeability of nanocomposite. The higher the diffusion coefficient is, the better the water vapor permeability is. To study the diffusion coefficient of H_2_O in films, the MSDs of H_2_O in the films were analyzed (Figure 14). Diffusivity was calculated by using the slope of MSD diagram (Figure 14a) [55].

The results show that the diffusivity changes as the change of fillers (Figure 14b). The addition of SiO_2_ (modified-SiO_2_) nanoparticles improves the diffusivity of H_2_O molecules in PMA films. The addition of SiO_2_ nanoparticles results in bigger voids at the interface of PMA/SiO_2_ (modified-SiO_2_) film, which facilitates the rapid passage of H_2_O molecules. The diffusion coefficient of water molecules in PMA/KH550-SiO_2_ composite system is the best. And after that, the order of diffusion coefficient of water molecules in composite systems from high to low is PMA/KH560-SiO_2_, PMA/KH570, PMA and PMA/SiO_2_, respectively. This is consistent with the results of the water vapor permeability of the previous films.

## 4. Conclusions

In this study, experimental methods combined with molecular simulation ways have been successfully applied to study the microstructure-property relationship in various polyacrylate/modified-SiO_2_ composite systems. To investigate the effect of different surface structures on mechanical property and water vapor permeability of resultant PMA/SiO_2_ nanocomposite films, SiO_2_ particles were treated with polysiloxane (KH550, KH560 and KH570). Different functional groups on the surface of SiO_2_ lead to different interfacial interactions with PMA, which give different mechanical properties of composite films. At the same time, due to the different hydrophilic and hydrophobic surface of SiO_2_, water vapor permeability of composite film is different. It can be used to study the interfacial interaction and microstructure-property relationships of polyacrylate-based nanocomposites, thus guiding the design of high performance polyacrylate-based nanocomposites.

## Figures and Tables

**Table 1:** Thermal data of the composite films from TGA analysis.

Samples	Weight Loss Temperature (°C)		Heat-Resistance Index ^a^ (°C)
	T_5_	T_30_	
0	318.43	375.43	172.79
1	325.42	374.92	174.01
2	327.88	375.88	174.77
3	332.99	374.49	175.37
4	329.98	376.98	175.51

0. PMA, 1. PMA/SiO_2_, 2. PMA/KH550-SiO_2_, 3. PMA/KH560-SiO_2_ and 4. PMA/KH570-SiO_2_. ^a^ Heat resistance index = 0.49[T_5_ + 0.6(T_30_ − T_5_)]; T_5_, T_30_ is the decomposing temperature at 5%, 30% weight loss, respectively.

**Table 2:** Binding energy of PMA/SiO_2_ and PMA/modified-SiO_2_ composites.

Systems	*E_total_* (kcal/mol)	*E_PMA_* (kcal/mol)	*E_Si_*_*O*_2__ (or *E_modified-Si_*_*O*_2__) (kcal/mol)	*E*_inter_ (kcal/mol)	*E_binding_* (kcal/mol)
PMA	10,043.97	10,043.97	-	-	-
PMA/SiO_2_	−1812.38	12,722.97	−14,260.52	−274.83	274.83
PMA/KH550-SiO_2_	−2323.97	12,545.98	−14,552.63	−317.32	317.32
PMA/KH560-SiO_2_	−381.20	13,785.56	−13,810.49	−356.27	356.27
PMA/KH570-SiO_2_	−1646.98	-	-	-	-

## Supplementary Materials

The following are available online at https://www.mdpi.com/2073-4360/12/1/170/s1, Figure S1: Models for water diffusion in composite system, Figure S2. Models for MD simulation of composite system, Table S1: Binding energy of PMA/SiO_2_ and PMA/KH560-SiO_2_ composites, Table S2: Binding energy of composites system.

## References

[1] Lu Y., Ji H., Zhu K., Jing W., Qiu J. (2016). Dramatically improved piezoelectric properties of poly(vinylidene fluoride) composites by incorporating aligned TiO_2_ @MWCNTs. Compos. Sci. Technol..

[2] Wang H., Hsu J.H., Yi S.I., Kim S.L., Choi K., Yang G., Yu C. (2016). Thermally Driven Large N-Type Voltage Responses from Hybrids of Carbon Nanotubes and Poly(3,4-ethylenedioxythiophene) with Tetrakis(dimethylamino)ethylene. Adv. Mater..

[3] Yu C., Zhang H.B., Yang Y., Mu W., Cao A., Yu Z.Z. (2016). High-Performance Epoxy Nanocomposites Reinforced with Three-Dimensional Carbon Nanotube Sponge for Electromagnetic Interference Shielding. Adv. Funct. Mater..

[4] Hou G., Tao W., Liu J., Gao Y., Zhang L., Li Y. (2017). Tailoring the dispersion of nanoparticles and the mechanical behavior of polymer nanocomposites by designing the chain architecture. Phys. Chem. Chem. Phys..

[5] Shojaeiarani J., Bajwa D., Stark N.M. (2018). Spin-coating: A new approach for improving dispersion of cellulose nanocrystals and mechanical properties of poly (lactic acid) composites. Carbohyd. Polym..

[6] Yue Y., Xu M., Hai P., Ren D., Li K., Liu X. (2018). Secondary dispersion of BaTiO_3_ for the enhanced mechanical properties of the Poly (arylene ether nitrile)-based composite laminates. Polym. Test..

[7] Kazemi Y., Kakroodi A.R., Ameli A., Filleter T., Park C.B. (2018). Highly stretchable conductive thermoplastic vulcanizate/carbon nanotube nanocomposites with segregated structure, low percolation threshold and improved cyclic electromechanical performance. J. Mater. Chem. C.

[8] Arjmand M., Sadeghi S., Khajehpour M., Sundararaj U. (2016). Carbon nanotube/graphene nanoribbon/polyvinylidene fluoride hybrid nanocomposites: Rheological and dielectric properties. J. Phys. Chem. C.

[9] Kou Z., Zhang L., Ma Y., Liu X., Zang W., Zhang J., Huang S., Du Y., Cheetham A.K., Wang J. (2019). 2D carbide nanomeshes and their assembling into 3D microflowers for efficient water splitting. Appl. Catal. B Environ..

[10] Si Y., Huang T., Li Q., Huang Y., Gao S., Chen M., Wu L. (2018). Hierarchical Macro–Mesoporous Polymeric Carbon Nitride Microspheres with Narrow Bandgap for Enhanced Photocatalytic Hydrogen Production. Adv. Mater. Interfaces.

[11] Senses E., Akcora P. (2013). An Interface-Driven Stiffening Mechanism in Polymer Nanocomposites. Macromolecules.

[12] Yang J., Han C.R., Duan J.F., Xu F., Sun R.C. (2013). Interaction of Silica Nanoparticle/Polymer Nanocomposite Cluster Network Structure: Revisiting the Reinforcement Mechanism. J. Phys. Chem. C.

[13] Petcu C., Purcar V., Spătaru C., Alexandrescu E., Şomoghi R., Trică B., Niţu S., Panaitescu D., Dan D., Jecu M.L. (2017). The Influence of New Hydrophobic Silica Nanoparticles on the Surface Properties of the Films Obtained from Bilayer Hybrids. Nanomaterials.

[14] Sabzi M., Mirabedini S.M., Zohuriaan-Mehr J., Atai M. (2009). Surface modification of TiO_2_ nano-particles with silane coupling agent and investigation of its effect on the properties of polyurethane composite coating. Prog. Org. Coat..

[15] Kumar S.K., Jouault N., Benicewicz B., Neely T. (2013). Nanocomposites with Polymer Grafted Nanoparticles. Macromolecules.

[16] Zhang X., Zheng J., Fang H., Zhang Y., Bai S., He G. (2018). High dimensional stability and low viscous response solid propellant binder based on graphene oxide nanosheets and dual cross-linked polyurethane. Compos. Sci. Technol..

[17] Zou Y., Sun Y., He J., Tang Z., Zhu L., Luo Y., Liu F. (2016). Enhancing mechanical properties of styrene-butadiene rubber/silica nanocomposites by in situ interfacial modification with a novel rare-earth complex. Compos. Part A Appl. Sci. Manuf..

[18] Fang H., Zhao Y., Zhang Y., Ren Y., Bai S. (2017). Three-dimensional graphene foam-filled elastomer composites with high thermal and mechanical properties. ACS Appl. Mater. Inter..

[19] Zhang S., Meng G., Chen Z., Qiu H.L., Liu X. (2014). Grafting photosensitive polyurethane onto colloidal silica for use in UV-curing polyurethane nanocomposites. Colloids Surf. A Physicochem. Eng. Asp..

[20] Yu Y., Shu Y., Ye L. (2018). In situ crosslinking of poly (vinyl alcohol)/graphene oxide-glutamic acid nano-composite hydrogel as microbial carrier: Intercalation structure and its wastewater treatment performance. Chem. Eng. J..

[21] Huang Q., Liu M., Chen J., Wan Q., Tian J., Huang L., Jiang R., Deng F., Wen Y., Zhang X. (2017). Marrying the mussel inspired chemistry and Kabachnik-Fields reaction for preparation of SiO_2_ polymer composites and enhancement removal of methylene blue. Appl. Surf. Sci..

[22] Chen Y., Lin A., Gan F. (2006). Improvement of polyacrylate coating by filling modified nano-TiO_2_. Appl. Surf. Sci..

[23] Zhao F., Zeng X., Li H., Zhang J. (2012). Preparation and characterization of nano-SiO_2_/fluorinated polyacrylate composite latex via nano-SiO_2_/acrylate dispersion. Colloids Surf. A Physicochem. Eng. Asp..

[24] Gao D., Rui C., Lyu B., Ma J., Duan X. (2018). Preparation of epoxy-acrylate copolymer/nano-silica via Pickering emulsion polymerization and its application as printing binder. Appl. Surf. Sci..

[25] Yan B., Shi C., Ma J., Bing W., Zhang Y. (2015). Double in-situ synthesis of polyacrylate/nano-TiO_2_ composite latex. Prog. Org. Coat..

[26] Gao D., Chang R., Lyu B., Ma J. (2019). Growth from spherical to rod-like SiO_2_: Impact on microstructure and performance of nanocomposite. J. Alloys Compd..

[27] Gao D., Chang R., Lyu B., Ma J., Liu J., Li Q. (2019). Synthesis of raspberry-like SiO_2_/polyacrylate nanocomposite latexes via a one-step miniemulsion polymerization and its film properties. J. Sol-Gel Sci. Technol..

[28] Ma J., Hu J., Zhang Z. (2007). Polyacrylate/silica nanocomposite materials prepared by sol–gel process. Eur. Polym. J..

[29] Bao Y., Shi C., Yang Y., Ma J., Sha R. (2015). Effect of hollow silica spheres on water vapor permeability of polyacrylate film. RSC Adv..

[30] Lu J., Liu D., Yang X., Zhao Y., Liu H., Tang H., Cui F. (2015). Molecular dynamics simulations of interfacial interactions between small nanoparticles during diffusion-limited aggregation. Appl. Surf. Sci..

[31] Roussou R., Karatasos K. (2016). Graphene/poly(ethylene glycol) nanocomposites as studied by molecular dynamics simulations. Mater. Des..

[32] Wei Q., Zhang Y., Wang Y., Yang M. (2017). A molecular dynamic simulation method to elucidate the interaction mechanism of nano-SiO_2_ in polymer blends. J. Mater. Sci..

[33] Sadeghi S., Arjmand M., Otero Navas I., Zehtab Yazdi A., Sundararaj U. (2017). Effect of nanofiller geometry on network formation in polymeric nanocomposites: Comparison of rheological and electrical properties of multiwalled carbon nanotube and graphene nanoribbon. Macromolecules.

[34] Gooneie A., Schuschnigg S., Holzer C. (2016). Dissipative Particle Dynamics Models of Orientation of Weakly-Interacting Anisometric Silicate Particles in Polymer Melts under Shear Flow: Comparison with the Standard Orientation Models. Macromol. Theor. Simul..

[35] Gooneie A., Hufenus R. (2018). Hybrid carbon nanoparticles in polymer matrix for efficient connected networks: Self-assembly and continuous pathways. Macromolecules.

[36] Majidian M., Grimaldi C., Forró L., Magrez A. (2017). Role of the particle size polydispersity in the electrical conductivity of carbon nanotube-epoxy composites. Sci. Rep..

[37] Fujishiro S., Kan K., Akashi M., Ajiro H. (2017). Stability of adhesive interfaces by stereocomplex formation of polylactides and hybridization with nanoparticles. Polym. Degrad. Stab..

[38] Rissanou N.A., Power J.A., Harmandaris V. (2015). Structural and Dynamical Properties of Polyethylene/Graphene Nanocomposites through Molecular Dynamics Simulations. Polymers.

[39] Wang Y., Wang W., Zhang Z., Xu L., Li P. (2016). Study of the glass transition temperature and the mechanical properties of PET/modified silica nanocomposite by molecular dynamics simulation. Eur. Polym. J..

[40] Wei L., Zhang W., Ma J., Bai S., Ren Y., Liu C., Simion D., Qin J. (2019). π-π stacking interface design for improving the strength and electromagnetic interference shielding of ultrathin and flexible water-borne polymer/sulfonated graphene composite. Carbon.

[41] Dai S., Yan L., Huang Z., Zhao X. (2017). Molecular dynamics simulations on the interaction between microsphere and water in nanosilica/crosslinked polyacrylamide microsphere aqueous solution with a core–shell structure and its swelling behavior. Compos. Interface.

[42] Dai S., Yan L., Zhang J., Zhang T., Huang Z., Zhao X. (2017). Molecular dynamic simulation of core–shell structure: Study of the interaction between modified surface of nano-SiO_2_ and PAMAA in vacuum and aqueous solution. Compos. Interface.

[43] Colmenero J., Alvarez F., Arbe A. (2002). Self-motion and the alpha relaxation in a simulated glass-forming polymer: Crossover from Gaussian to non-Gaussian dynamic behavior. Phys. Rev. E Stat. Nonlin. Soft Matter. Phys..

[44] Basconi J.E., Shirts M.R. (2013). Effects of Temperature Control Algorithms on Transport Properties and Kinetics in Molecular Dynamics Simulations. J. Chem. Theory Comput..

[45] Davoodi J., Ahmadi M. (2012). Molecular dynamics simulation of elastic properties of CuPd nanowire. Compos. Part B Eng..

[46] Martín-Fabiani I., Koh M.L., Dalmas F., Elidottir K.L., Hinder S.J., Jurewicz I., Lansalot M., Bourgeat-Lami E., Keddie J.L. (2018). Design of waterborne nanoceria/polymer nanocomposite UV-absorbing coatings: Pickering versus blended particles. ACS Appl. Nano Mater..

[47] Zhang L., Luo M., Sun S., Ma J., Li C. (2010). Effect of Surface Structure of Nano-CaCO_3_ Particles on Mechanical and Rheological Properties of PVC Composites. J. Macromol. Sci. Part B.

[48] Meijer H.E.H., Govaert L.E. (2005). Mechanical performance of polymer systems: The relation between structure and properties. Prog. Polym. Sci..

[49] Jancar J., Kucera J. (2004). Yield behavior of polypropylene filled with CaCO_3_ and Mg(OH)_2_. I. ‘Zero’ interfacial adhesion. Polym. Eng. Sci..

[50] Shen J., Sun J.W., Hu Y., Kan C.Y. (2017). Polysiloxane/polyacrylate composite latexes with balanced mechanical property and breathability: Effect of core/shell mass ratio. J. Appl. Polym. Sci..

[51] Yan B., Yang Y., Ma J. (2013). Fabrication of monodisperse hollow silica spheres and effect on water vapor permeability of polyacrylate membrane. J. Colloid Interface Sci..

[52] Huangfu Y., Ruan K., Qiu H., Lu Y., Liang C., Kong J., Gu J. (2019). Fabrication and investigation on the PANI/MWCNT/thermally annealed graphene aerogel/epoxy electromagnetic interference shielding nanocomposites. Compos. Part A: Appl. Sci. Manuf..

[53] Yang X., Tang L., Guo Y., Liang C., Zhang Q., Kou K., Gu J. (2017). Improvement of thermal conductivities for PPS dielectric nanocomposites via incorporating NH_2_-POSS functionalized nBN fillers. Compos. Part A Appl. Sci. Manuf..

[54] Song M., Zhao X., Li Y., Hu S., Zhang L., Wu S. (2014). Molecular dynamics simulations and microscopic analysis of the damping performance of hindered phenol AO-60/nitrile-butadiene rubber composites. RSC Adv..

[55] Khosravanian A., Dehghani M., Pazirofteh M., Asghari M., Mohammadi A.H., Shahsavari D. (2018). Grand canonical Monte Carlo and molecular dynamics simulations of the structural properties, diffusion and adsorption of hydrogen molecules through poly (benzimidazoles)/nanoparticle oxides composites. Int. J. Hydrogen Energy.

