# Supplementary material for: Surface Modification Design for Improving the Strength and Water Vapor Permeability of Waterborne Polymer/SiO2 Composites: Molecular Simulation and Experimental Analyses
Source: Polymers (Basel). 2020 Jan 9;12(1):170. doi: 10.3390/polym12010170 (PMC7023158; doi:10.3390/polym12010170)
Supplement: Supplementary file 1 [file polymers-12-00170-s001.pdf]

Supplementary Information

Article

Surface Modification Design for Improving the Strength and Water Vapor Permeability of Waterborne Polymer/SiO_2 Composites: Molecular Simulation and Experimental Analyses

Yingke Wu [1], Jianzhong Ma [2,*], Chao Liu [3,*] and Hongxia Yan [4]

1 School of Materials Science & Engineering, Shaanxi University of Science and Technology, Xi'an 710021, China; einske@163.com
2 Key Laboratory of Leather Cleaner Production, China National Light Industry, College of Bioresources Chemical and Materials Engineering, Shaanxi University of Science and Technology, Xi'an 710021, China
3 Shaanxi Collaborative Innovation Center of Industrial Auxiliary Chemistry and Technology, Xi'an 710021, China
4 Department of Applied Chemistry, School of Science, Northwestern Polytechnical University, Xi'an 710129, China; hongxiayan@nwpu.edu.cn
* Correspondence: majz@sust.edu.cn (J.M.); lc1010158@163.com (C.L.); Tel.: +86-029-86132559 (ext. 601) (J.M.)

1. Construct the composite system containing water model

To study the diffusion coefficient of H_2O in composite systems, the MSDs of H_2O in composite systems were analyzed. Some composite systems containing water molecules were constructed as follows (Figure S1):

PMA/SiO_2 (KH550-SiO_2, KH560-SiO_2)/H_2O: Amorphous cells containing composites of PMA polymer chains with 20 repeat units, one SiO_2 (or modified-SiO_2) nanoparticle (diameter 20 nm) and 10 H_2O molecules were constructed, and periodic boundary conditions were applied.

PMA/KH570-SiO_2/H_2O: Amorphous cells containing composites of PMA polymer chains with 19 repeat units, one PMA-KH570-SiO_2 and 10 H_2O molecules were constructed, and periodic boundary conditions were applied.

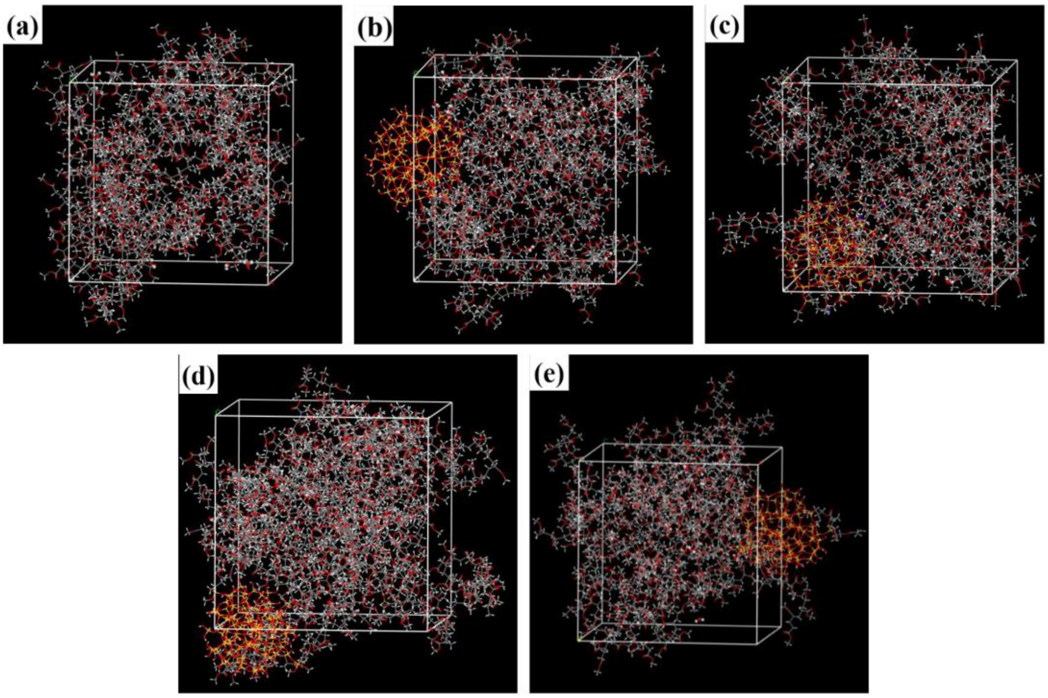

Figure S1. Models for water diffusion in composite system: **a.** PMA/H_2O, **b.** PMA/SiO_2/H_2O, **c.** PMA/KH550-SiO_2/H_2O, **d.** PMA/KH560-SiO_2/H_2O, and **e.** PMA/KH570-SiO_2/H_2O.

2. Binding energy of PMA/SiO_2 and $PMA/modified\text{-}SiO_2$ composites (30 PMA polymer chains)

A system of 30 PMA polymer chains and one SiO_2 was re-simulated, mainly hoping to get consistent trends with experimental results (Figure S2). The simulation calculation of PMA/SiO_2 and PMA/KH550-SiO_2 nanocomposite system has been completed, and the results are as follows (Table S1 and Table S2):

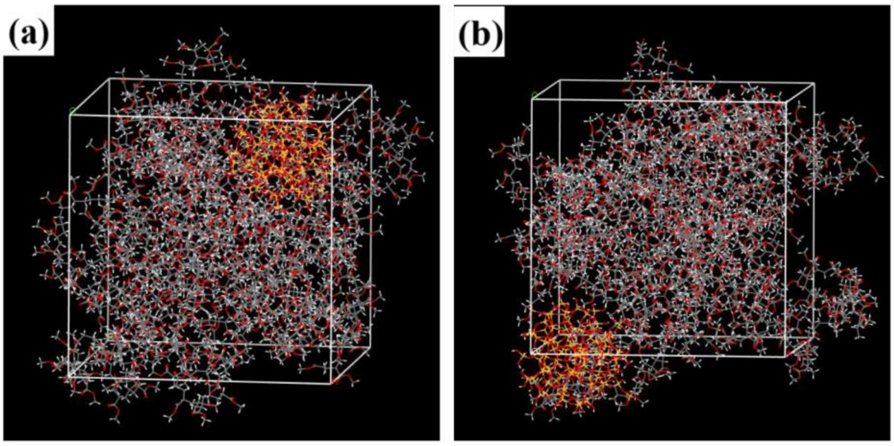

Figure S2. Models for MD simulation of composite system: **a.** PMA/SiO_2, **b.** PMA/KH560-SiO_2.

Table S1. Binding energy of PMA/SiO_2 and PMA/KH560-SiO_2 composites (30 PMA polymer chains).

Systems	E_{total} （kcal/mol）	E_{PMA} （kcal/mol）	E_{SiO2}(or $E_{modified\text{-}SiO2}$) (kcal/mol)	E_{inter} (kcal/mol)	$E_{binding}$ (kcal/mol)
PMA/SiO_2	2939.02	17572.19	-14344.23	-288.93	288.93
PMA/KH560-SiO_2	3919.87	18219.59	-13957.74	-341.98	341.96

Table S2. Binding energy of composites system (20 PMA polymer chains and 30 PMA polymer chains).

Systems	$E_{binding}$ (kcal/mol)
PMA/SiO_2 (20 PMA polymer chains)	274.83
PMA/SiO_2 (30 PMA polymer chains)	288.93
PMA/KH560-SiO_2 (20 PMA polymer chains)	356.27
PMA/KH560-SiO_2 (30 PMA polymer chains)	341.96

The simulation results are in line with the experimental results and are consistent with the simulation results in the manuscript.
